# Investigating the interplay of loneliness, computer-mediated communication, online social capital, and well-being: insights from a COVID-19 lockdown study

**DOI:** 10.3389/fdgth.2024.1289451

**Published:** 2024-06-14

**Authors:** Megan Fahy, Marguerite Barry

**Affiliations:** University College Dublin, Dublin, Ireland

**Keywords:** social capital, wellbeing, computer-mediated communication, technology, loneliness

## Abstract

**Introduction:**

Recent studies have found that there is scope for communication technologies to increase online social capital. Although studies have linked online social capital and mental well-being, there is a need to identify the causal pathways within this relationship. This study explores the role of loneliness in the relationship between computer-mediated communication, online social capital and well-being.

**Methods:**

The study used an online questionnaire and had 217 participants. William's 2006 scale was used to measure individuals’ online social capital, and structural equational modelling (SEM) was used to explore the relationship between computer-mediated communication, use, levels of loneliness, online social capital and well-being. This study was conducted remotely during the first COVID-19 lockdown in Ireland.

**Results:**

High levels of online communication mitigated the otherwise negative effects of loneliness on well-being when online interaction fostered online social capital.

**Conclusion:**

Overall, the proposed model offers qualified support for the continued analysis of technology-mediated communication as a potential source for building online social capital and improving the well-being of particular individuals with high levels of loneliness.

## 1 Introduction

This study recognises that the relationship between technology and well-being is heterogeneous and nuanced, often characterised by diverse findings and theoretical debate. The primary motivation behind this study is to delve deeper into the interplay between computer-mediated communication, social capital, loneliness, and well-being. While existing studies have offered valuable insights, they often focus on specific technologies or platforms, limiting the generalisability of findings, e.g., focusing entirely on social networking (1), or have focused on general use of the internet rather than communicative uses (2). The complex nature of well-being and the diverse ways individuals engage with technology necessitate a more comprehensive approach. In this introduction, we discuss the need to explore the underlying mechanisms that drive the relationship between technology use and well-being, including mediating factors and patterns of use. Additionally, this study aims to leverage advanced analytical methods suggested in previous research to provide a more comprehensive and nuanced exploration of these relationships.

This study scoped the limitations highlighted in related literature with conflicting results to address the complexities of studying technology use, social capital, loneliness, and well-being. A study by Arampatzi et al. found a negative association between the number of hours spent on social networking sites and the subjective well-being of users who experienced loneliness and dissatisfaction with their contacts. The study noted that those with low-quality social capital should avoid intensive use of social networking site platforms. This study noted a fundamental limitation; this advice could create a Catch-22 situation because social networking sites may be one of the only ways for these people to maintain contact with others. It suggested that future research should note how social media is utilised in terms of “scrolling” vs. “communication” (1). Another study also distinguished between the general use of technology and the use of technology focused on communication. This study focused on online gameplay, and technology use was linked with increased well-being, where social capital in games is strongly and positively related to players’ well-being when communication and interaction are critical components of technology utilisation (2). These two studies highlight a gap in the research where studies have not focused on computer-mediated communication vs. general internet use. In terms of analytics, one study that showed a positive link between computer-mediated communication, social capital, and well-being suggested that structural equational modelling be utilised to add a dimension to the enquiry process or other advanced methods such as partial least squares (3).

Jin examined the motivations behind the use of technology and how that impacts the relationship between social capital and well-being. It highlights that not all motivations for technology use may impact well-being in the same way, and the social context in which individuals engage in technology use can significantly shape these outcomes. The study mentions that it did not address a range of external motivating factors, such as the concept of self-satisfaction. For the purpose of this study, loneliness was considered an appropriate external motivating factor to investigate (4).

Mandryk et al. (5) noted how previous research focused on the harm caused by online gaming. They argued that the relationship between well-being and loneliness depends on contingent factors related to the player, the game, and the gaming context. They focused on the underlying dynamic of passion for playing (World of War Craft), and findings indicated that while harmonious passion fosters in-game social connections, alleviates loneliness, and enhances well-being, obsessive passion contributes to building social capital but is associated with increased loneliness. These results underscore the significance of considering underlying mechanisms such as passion orientation in understanding the relationship between gaming and well-being. The authors of this study noted the limitations of a population of gamers and also the use of the game-specific environment and game-specific features when it comes to linking well-being, loneliness and social capital through the use of technology, meaning that these constructs explored could be specific to a gaming environment and need to be researched in a broader scope of computer-mediated communication. A study of older adults found that “Line”, which focused on chatting and communication, moderated the effect between social capital and loneliness, while Facebook did not. The study noted that future studies should focus on “specific factors” to resolve this intriguing question of the relationship between technology use, social capital, well-being and loneliness (6). Studying “computer-mediated communication” allows explicitly the exploration of multifaceted relationships between technology use, social connections, loneliness, and well-being across different platforms and contexts.

This study aims to unravel the pathways between loneliness, social capital, well-being, and computer-mediated communication further by using a similar structural equational model as that used by Magsamen-Conrad et al. which showed that the presence of computers in human behaviours (and specifically, channels of communication) enables some individuals (particularly those prone to self-concealment) to communicate and foster social capital and improve their well-being (7). The significance of computer-mediated communication tools became apparent during the pandemic as they played a crucial role in maintaining social connections and mitigating potential risks to social health (8). COVID-19 offered a unique opportunity to understand how computer-mediated communication may foster digital social capital and promote well-being in a time of social isolation, so this study offers distinctive insights.

### 1.1 Loneliness and use of computer-mediated communication

A recent report revealed that Ireland has the highest levels of loneliness in Europe (9). Loneliness is a subjective feeling defined as an unmet need regarding the quantity or quality of social interactions. Being lonely is a negative *feeling* conceptually distinct from being alone (10). Pooled data by Van As et al. supported the longitudinal association between loneliness and mental well-being, in particular, depressive symptoms (11). A scoping review by Peterson et al. 2023, “The association between information and communication technologies, loneliness and social connectedness”, found that using information communication technologies (ICT) is associated with decreased loneliness and depression and increased well-being. The paper noted that the benefits of ICTs seem to be tied to strengthening pre-existing and new social connections and provided a starting point for future mediation and moderation analyses (12).

**Figure 1:**
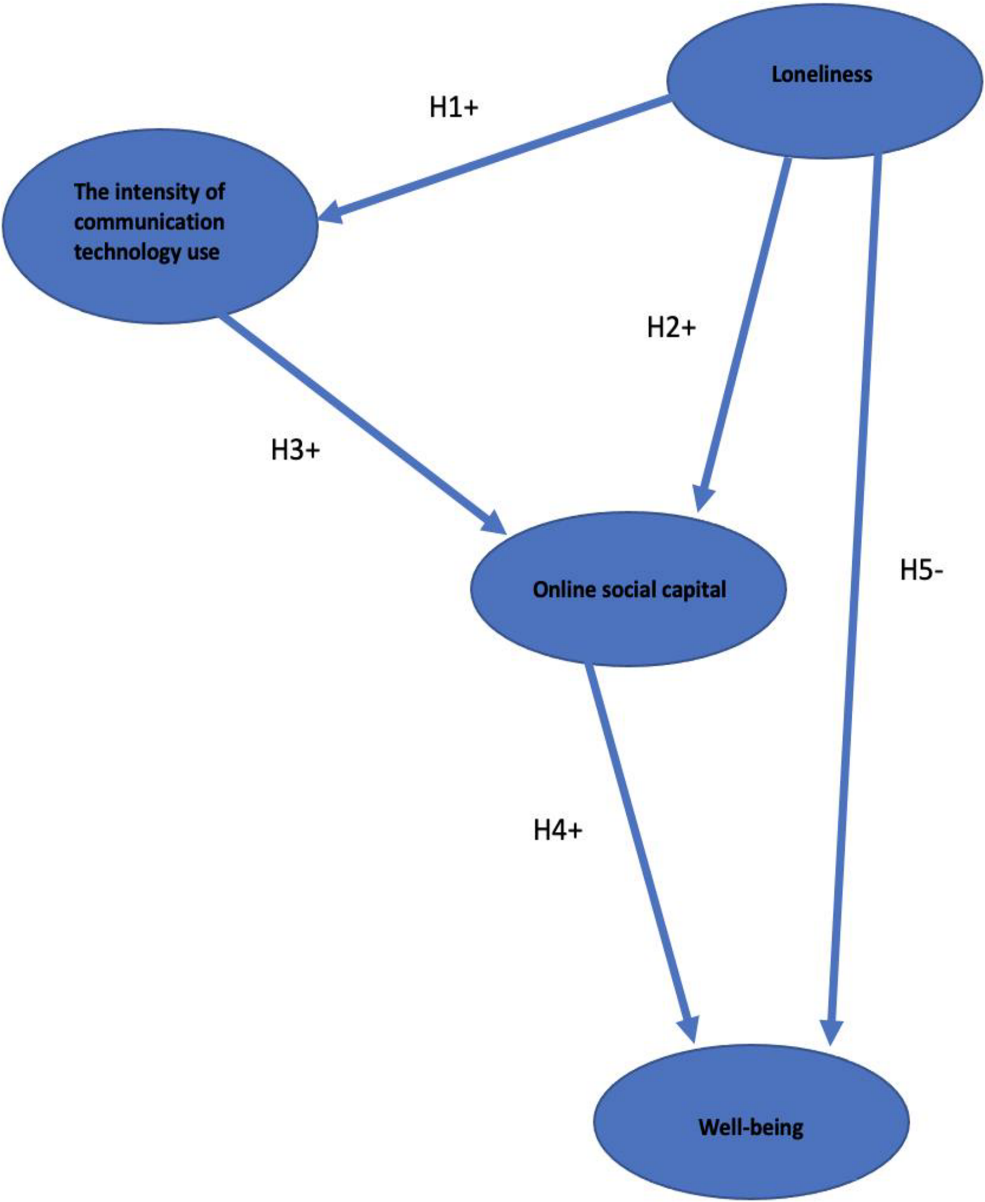
This figure shows the hypothesised model to examine how increased communication through online channels may ultimately have positive outcomes for well-being.

Nowland et al. proposed a theoretical model to explain the dynamic relationship between loneliness and social internet use. The model suggests that the connection between loneliness and internet use depends on how it is utilised. If the internet is used to make new connections or strengthen existing relationships, it can help reduce loneliness. However, if used to avoid social interactions and escape from the challenges of being around others, it can further contribute to loneliness (13). A hypothesised model was developed to examine how communication technologies may ultimately have positive outcomes for well-being (see Figure 1). Research has shown that loneliness is often associated with increased use of communication technologies to alleviate social isolation (11). Hence, hypothesis 1 [H1 hereafter] proposes that loneliness will predict higher levels of self-reported technology use. This hypothesis is grounded in the idea that individuals experiencing loneliness may be more inclined to seek connection and social interactions through technology platforms.

H1 represents the hypothesis that more loneliness will predict more self-reported technology use.

### 1.2 Online social capital

Social capital theory has existed since the mid-twentieth century. Social capital conceptualises how people connect through their social networks, develop common values within them (such as trust and reciprocity), and create a kind of resource (14). These social ties are vital because they provide social support, such as advice and companionship (15), valuable information, such as health information (16), and facilitate the resolution of collective action problems, such as small or large-scale protests (17).

In recent years, benefits derived from social uses of the internet have been broadly conceptualised as social capital outcomes (18, 19). In 2006, William's developed the internet-focused social capital research scale. Items on the scale that measure ‘bridging social capital’ focus on whether a person is outward-looking, has contact with a broad range of people, has a view of oneself as part of a wider group and has diffuse reciprocity with a broader community. An example of a question on this scale is, interacting with people online makes me feel like part of a larger group. The “bonding” scale measures emotional support, access to scarce or limited resources, and the ability to mobilise solidarity. An example of a question on this scale is community: “When I feel lonely, there are several people online I can talk to” (20). Some studies argue that technology enables broader, more effective social networks, supplementing social capital (21). H2, in turn, extends the hypothesis by suggesting that loneliness will also predict more digital social capital.

H2 anticipates that loneliness is a predictor not only for heightened technology use but also for developing digital social capital.

Researchers have found that online interactions may supplement in-person interactions, mitigating loss from time spent on technology (21). Therefore, as technology use increases, so will the levels of digital social capital.

H3 suggests that increased technology use will predict greater digital social capital.

### 1.3 Loneliness and wellbeing: the role of mediated communication

The accumulation of social capital is associated with many positive outcomes, including increased life satisfaction and better public health (22, 23), enhanced self-esteem, and general physical and psychological well-being (24, 25). Studies have demonstrated that people with higher levels of social capital have lower mortality rates and better self-report health status than people with lower levels of social capital (26), while stronger social ties have been associated with reducing adverse health outcomes ranging from depression to alcoholism (27) Recent studies have found that computer-mediated communication is positively associated with users’ formation and maintenance of digital social capital and is linked to positive well-being (28–31).

H4 posits that higher levels of digital social capital predict better mental well-being.

H5 introduces the direct relationship between loneliness and reduced well-being. This hypothesis is rooted in the well-established understanding that loneliness is a significant predictor of adverse mental health outcomes. Research has consistently shown that individuals experiencing loneliness are more likely to face diminished well-being (10, 32). Therefore,

H5 posits that loneliness directly predicts reduced well-being, independent of other factors.

In summary, H1-H4 collectively proposes a partially mediated relationship between loneliness and well-being, suggesting that individuals with higher levels of loneliness can still experience positive well-being through the intermediary role of digital social capital, even in the context of computer-mediated communication. The structural equational model drew on existing empirical evidence and theoretical foundations to support the expected relationships between loneliness, technology use, digital social capital, and well-being.

Figure 1 shows the hypothesised model to examine how increased communication through online channels may ultimately have positive outcomes for well-being. Within the model, several arrows explain the relationships between the concepts discussed through the literature and analysed within the study. First, the arrow H1+ represents the hypothesis that more loneliness will predict more computer-mediated communication, use and more online social capital (H2+). Second, the H3+ arrow represents the hypothesis that more computer-mediated communication, use will predict more online social capital. Third, the H4+ arrows represent the hypothesis that more online social capital predicts better well-being. Finally, the H5- arrow represents the hypothesis that higher levels of loneliness predict reduced well-being. That is, H5 represents the direct relationship between loneliness and well-being, such that those who are more lonely suffer from reduced experience of well-being. H1-H4 represent the partially mediated relationship between loneliness and well-being, such that individuals prone to loneliness can still experience positive well-being if driven to online mechanisms for communication and relationship building, relationship maintenance, social support and other aspects of online social capital.

## 2 Methods

### 2.1 Participants

217 participants completed the survey. All participants were over 18 years of age, with an age range of (*M *= 31). The gender distribution consisted of 210 participants (39%) identifying as male and 145 (61%) as female. Recruitment took place through social media platforms, specifically Twitter and Facebook. Initial testing involving 20 participants informed necessary adaptations. A power analysis determined the appropriate sample size for detecting the expected effects with adequate statistical power. Based on previous research and effect size estimates, a minimum sample size of 150 participants was required to achieve a power of 0.80 at an alpha level of 0.05. This sample size accounts for potential attrition and ensures sufficient statistical power to detect meaningful effects.

#### 2.1.1 Exclusion criteria

The study of well-being may involve content or questions that could be deemed inappropriate or challenging for minors. Obtaining parental consent for minors can be a complex process, introducing additional administrative burdens and potential barriers to participation. Excluding individuals under 18 streamlines the consent process and ensures that parental involvement is appropriately managed. This was a difficulty during COVID-19 when research had to occur exclusively online. While excluding individuals under 18 may have limited the generalisability of findings to this age group, it was a pragmatic decision that helped researchers focus on a population for which legal, ethical, and privacy considerations are more straightforward. The results for a younger population might offer interesting or contrasting insights, and it is recommended that this population be considered in future research.

### 2.2 Study context

The survey, a web-based questionnaire, was exclusively accessible online. The study occurred during the first COVID-19 lockdown in Ireland, a period marked by stringent restrictions to curb the spread of the virus. Participants were required to adhere to national guidelines, refrain from unnecessary travel, and limit outdoor activities to within 2 km of their residences. Notably, exceptions were granted for essential workers. The decision to conduct an online survey was based on the appropriateness of respondents’ ability to utilise computer-mediated communication successfully during this time.

### 2.3 Measures

Social capital was measured using The “On and Off the Net”: Scales by William (20). This was identified in a scoping review as the most validated and used measure for online social capital. Well-being was measured using the Warwick-Edinburgh Mental Well-being Scale, 2007 (30). Other variables measured included loneliness and internet utilisation. There were 30 questions to respond to, and this survey section took around 20 min to complete. All participants were asked to complete a participation form and given a brief outline of the purpose and scope of the study. These measures were picked as they were considered a good fit for the objective and goals of the study. All measurements were validated in previous research in a similar field.

#### 2.3.1 Well-being

The Warwick-Edinburgh Mental Well-being Scale (WEMWBS) (33) was employed in this study, consisting of 14 questions answered on a 5-point scale (See Appendix). Confirmatory Factor Analysis indicated that 11 out of the 14 statements were connected to a single underlying aspect of well-being. The model fit statistics revealed a Chi-square (*χ*^2^) of *χ*^2^(30) = 35.82, with a relative Chi-Square (relative *χ*^2^) of 2.42, indicating a statistically significant but reasonable fit (*p* = 0.001). The Comparative Fit Index (CFI) was.96, and the Root Mean Square Error of Approximation (RMSEA) was .07. These statistics collectively suggest that the model fits the data reasonably well. The average well-being score was 2.97 (on a scale of 1–5) with a Standard Deviation (SD) of .61. The reliability coefficient (α) was high at.89, indicating strong internal consistency.

#### 2.3.2 Loneliness

Loneliness was assessed using Russell's UCLA 20-item loneliness scale (revised version) (Russell et al.) (34). Participants responded on a 4-point scale, with an average loneliness score of 2.7 and a Standard Deviation (SD) of .75. The reliability coefficient (α) was .85, indicating good internal consistency.

#### 2.3.3 Social capital

Online social capital was measured using an adapted version of William's 2006 Scale, the Internet Social Capital Scale (ISCS) (Williams) (20), a revised 20-item scale based on Cohen and Hoberman's model (7). The model fit statistics for this scale indicated a Chi-square (*χ*^2^) of *χ*^2^(8) = 17.82, with a relative Chi-Square (relative *χ*^2^) of 2.42 (*p* = 0.01). The CFI was .98, and the RMSEA was .07, suggesting a good fit. The average social capital score was (*M* = 2.87) on a scale of 1–5, with a Standard Deviation (SD) of .91. The reliability coefficient (α) was .86, indicating high internal consistency. These statistical findings support the reliability and validity of the measurement scales used in the study.

#### 2.3.4 The utilisation of computer-mediated communication

Computer-mediated communication was systematically assessed through a carefully crafted set of three questions designed to capture participants’ engagement across various platforms and frequencies comprehensively. Participants were questioned on the forms of computer-mediated communication they utilised, including social media (e.g., Instagram, Facebook, TikTok, Twitter, etc.), text messages, messaging services (e.g., Facebook Messenger, WhatsApp, Signal, etc.), video conferencing tools (Zoom, Slack, Google Meets), internet forums, calling and Facetime. This list of platforms was carefully selected through a developmental process involving scale creation, pre-testing, and iterative revisions based on valuable participant feedback. Respondents could indicate their usage across the following options.

Weekly Usage Frequency: Participants were further probed on their computer-mediated communication engagement throughout the week. Responses were elicited on a scale ranging from 1 (not at all) to 5 (quite often), allowing for nuanced distinctions in participants’ weekly engagement levels. Respondents were prompted to indicate their daily usage frequency to capture the granularity of participants’ interaction with computer-mediated communication; this question utilised a scale ranging from 1 (not at all) to 5 (quite often), enabling the delineation of daily patterns in participants’ technology use.

## 3 Results

Using AMOS 22.0, the hypothesis presented was examined with the maximum likelihood SEM (structural equational model). This methodology considers the presence of measurement errors within the data, enabling practical evaluation. Per the recommended ‘two-step approach’ guidelines by Gerbing and Hunter (35) for structural equational modelling in practice, a confirmatory analysis was conducted on multi-item scales, assessing face validity and internal consistency. Reliability was gauged using Cronback’s alphas. The goodness of fit indices were used. The *X*^2^/*df* statistics were utilised to adjust the *X*^2^ value for sample size according to the principles and practices of SEM (Kline). The Root Mean Square Error of Approximation (RMSEA) considered approximation errors in the broader population. In accordance with Kline (36), the criteria for determining the appropriateness of model fit were as follows: A relative *X*^2^ value less than 3, CFI greater than 0.90, RMSEA less than 0.10.

**Figure 2:**
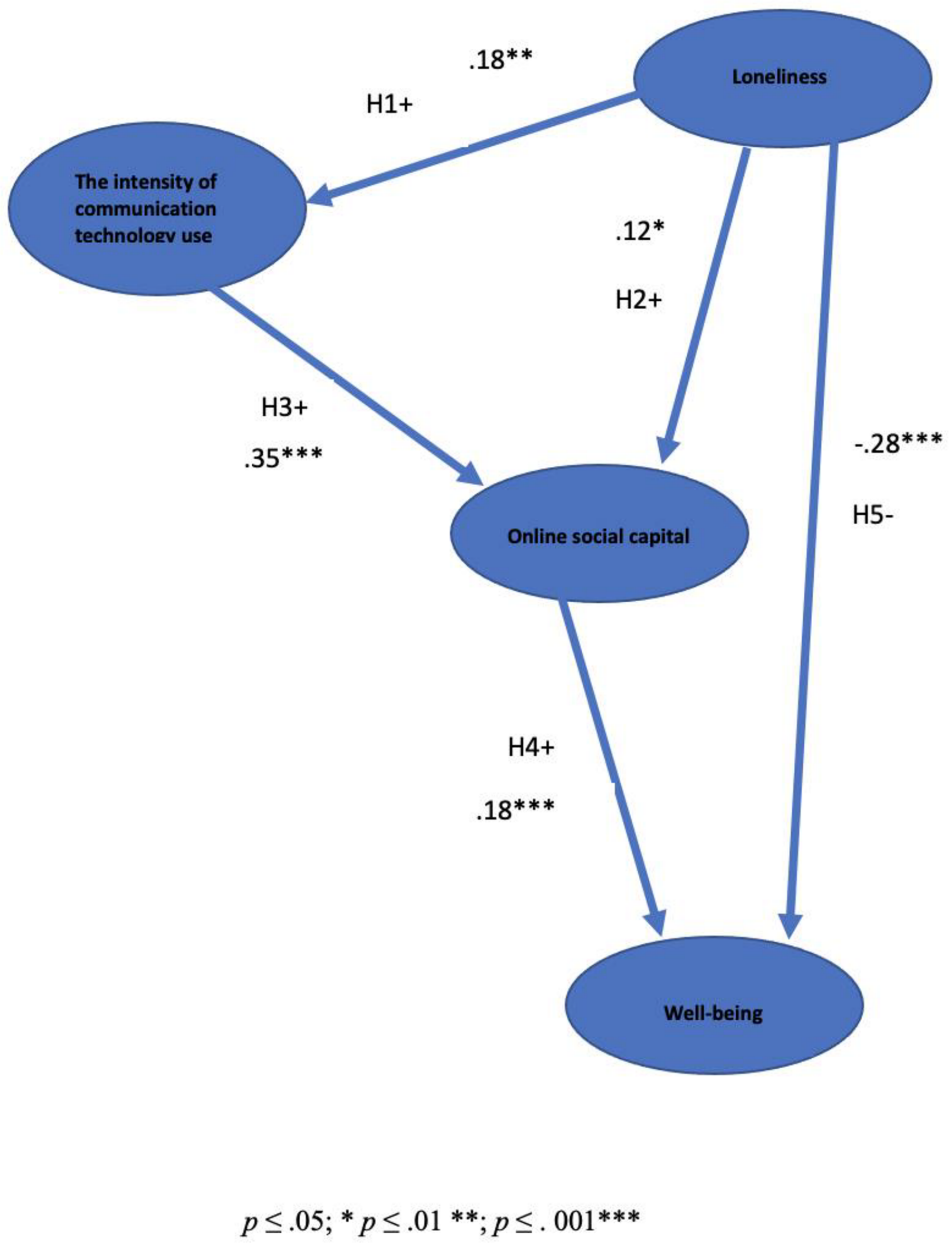
This figure shows the structural equational model supports the causal pathways and five hypotheses predicted.

The SEM model outcomes supported the proposed hypothesis concerning the relationship between technology use, loneliness, digital social capital and well-being (see Figure 2). Loneliness predicted more self-reported technology use. Loneliness also predicted digital social capital. Increased technology use predicted greater digital social capital. Higher levels of digital social capital predicted better mental well-being. Loneliness directly predicted reduced well-being, independent of other factors.

## 4 Discussion

Unravelling the Relationship Between Computer-mediated Communication, Use, Digital Social Capital and Wellbeing.

This study found that online social capital fostered through computer-mediated communication can positively impact people with high levels of loneliness. This supports recent studies that have found that computer-mediated communication is positively associated with users’ formation and maintenance of digital social capital and is linked to positive well-being (28–31). In their study conducted in 2004, Kawamura and Frost discovered that cultivating digital social capital, primarily through social networking sites and a feeling of belonging to an online community, can have beneficial effects. Particularly on the well-being of individuals who are shy, depressed, introverted, or experience difficulties in communication (37). This study suggests that this also extends to individuals with high levels of loneliness.

This research used a structural equational model to examine a causal model of the effects of loneliness on well-being with computer-mediated communication, use and online social capital as intervening factors (using the Handbook of Structural Equation M*odelling* by Hoyle) (38). The structural equational model supports the causal pathways and five hypotheses predicted in Figure 1. While high levels of loneliness are associated with reduced well-being, the SEM model indicates that when the causal paths between loneliness and well-being are mediated through computer-mediated communication and online social capital, the result is higher levels of well-being.

Research has consistently shown that individuals experiencing loneliness are more likely to face diminished well-being (10, 32). The implications of more frequent use of online-mediated communication can mediate the typically negative causal association between loneliness and well-being. That is, although lonely individuals may be driven to online channels of communication, as long as the outcome of the communication is fostering social capital, that is, the eight dimensions of social capital measured in William's scale for online social capital (outward-looking from usual daily existence, contact with a broader range of people, a view of oneself as part of a wider group, diffuse reciprocity with a broader community, emotional support, access to scarce or limited resources, ability to mobilise solidarity and out-group antagonism) increased well-being benefits can be achieved.

### 4.1 Practical implications for health promotion

The findings of this study have significant implications for health promotion strategies, particularly in addressing loneliness and enhancing wellbeing through computer-mediated communication. Health promotion initiatives can leverage these findings to design interventions that facilitate meaningful online interactions and foster digital social capital among individuals experiencing loneliness.

### 4.2 Limitations

While the significance of online connections became evident during the national lockdown, it is crucial to acknowledge the potential trade-off: increased reliance on digital communication might diminish opportunities for face-to-face interactions outside of lockdown scenarios. During the lockdown, there may have been self-reporting bias as participants may have been susceptible to overstating or understating their mental well-being due to factors such as heightened anxiety, social desirability bias or difficulty recalling their mental well-being over time.

One notable limitation is the restricted generalisability of our findings to minors. By excluding individuals under 18, our study primarily focuses on adults and may not capture essential nuances or challenges related to well-being and technology use in younger populations. Future research targeting minors would be necessary to understand well-being across different age groups comprehensively.

### 4.3 Suggestions for future research

The results of this study are particularly impactful for a subset of the population that experiences high levels of loneliness. There is a well-established link between personality and levels of loneliness (39, 40). There has been a call for a re-examination of the influence of the individual personality on Internet use and how it may both harm and benefit personalities on an individual level (41). This research furthers this cause by suggesting that individual differences may account for the contrasting impacts of internet use on well-being (42). There may be complexities at play that can be established by testing causal pathways. The structural equational model used in this study was useful, and it is recommended for future research as it allows researchers to test competing hypotheses and refine theoretical models.

It is recommended that further examination of the potential benefits of computer-mediated communication be explored using a mixed-methods approach to identify the complexities of these relationships and dynamics and enrich our understanding of internet use on a deeper level to understand implications for mental well-being.

A recent report revealed that Ireland has the highest levels of loneliness in Europe (9). It would be valuable to explore the SEM model design in other populations, such as communities facing acute and chronic loneliness, including migrants (43), individuals with disabilities or chronic illnesses (44), and communities affected by natural disasters (45). This could provide valuable insights into who could benefit from this research.

There is potential to explore the technological features that impact the casualty between variables. Interdisciplinary collaboration between researchers in psychology, sociology, communication and technology is recommended to study this area comprehensively.

This study was conducted during a unique and unprecedented national lockdown. As mentioned, this may have led to some self-reporting bias; however, this snapshot in time may also offer clues about how individuals utilised computer-mediated communication in a time of enforced isolation. It is recommended that this study be conducted again outside of these circumstances to provide comparison and further insight.

## 5 Conclusion

Overall, the proposed model offers qualified support for the continued analysis of technology-mediated communication as a potential source for improving the well-being of particular individuals with high levels of loneliness.

## Data Availability

The raw data supporting the conclusions of this article will be made available by the authors, without undue reservation.
