# Supplementary material for: Investigating the interplay of loneliness, computer-mediated communication, online social capital, and well-being: insights from a COVID-19 lockdown study
Source: Front Digit Health. 2024 Jun 14;6:1289451. doi: 10.3389/fdgth.2024.1289451 (PMC11211625; doi:10.3389/fdgth.2024.1289451)

**Appendix**

*Digital Social Capital and Well-being Survey – Participant Information Sheet and Consent Form*

Purpose of the Study: You are invited to participate in the Digital Social Capital and Well-being Survey, which aims to investigate the impact of communication technology on digital social capital and well-being. This survey seeks to contribute crucial insights into the evolving dynamics of social connections in the digital age and inform interventions to enhance well-being.

Your Involvement: Participation involves completing an anonymous survey consisting of survey questions, exploring your experiences related to communication technology, digital social capital, and well-being. Your responses will remain confidential, and you can opt not to answer any question without providing a reason. The survey is estimated to take approximately 15-20 minutes.

Eligibility: You are eligible to participate if you are over 18.

Voluntary Participation: Your involvement is entirely voluntary, and you can withdraw at any point without explanation. If you decide to participate, your responses will be submitted automatically as you progress, but you may terminate your involvement at any time.

Confidentiality: Your anonymity is paramount. No personally identifiable information will be collected, and a unique study ID will be assigned for data analysis, safeguarding your privacy. All survey data will be securely stored within University College Dublin on an encrypted facility, accessible only to approved researchers of the Digital Social Capital and Well-being Survey.

Data Handling: All survey data will be retained for a minimum of 10 years, adhering to the UCD Code of Research Conduct.

Results and Publication: The study's findings will be used to establish baseline information on the relationship between communication technology, digital social capital, and well-being. The results may be published in reports, and research journals, presented at conferences, or utilised in future research projects. Your identity will remain confidential.

Disadvantages and Withdrawal: No foreseeable negative consequences are expected from your participation. If you feel uncomfortable, you can withdraw at any time without repercussions.

Contact Information: Following completion of the survey, you can contact us via email) to discuss your experience or feelings. For health-related concerns, contact appropriate services.

Thank you for considering participation in the Digital Social Capital and Well-being Survey.

**Who has reviewed this study?** This study was approved by the Social Research Ethics Committee of UCD.


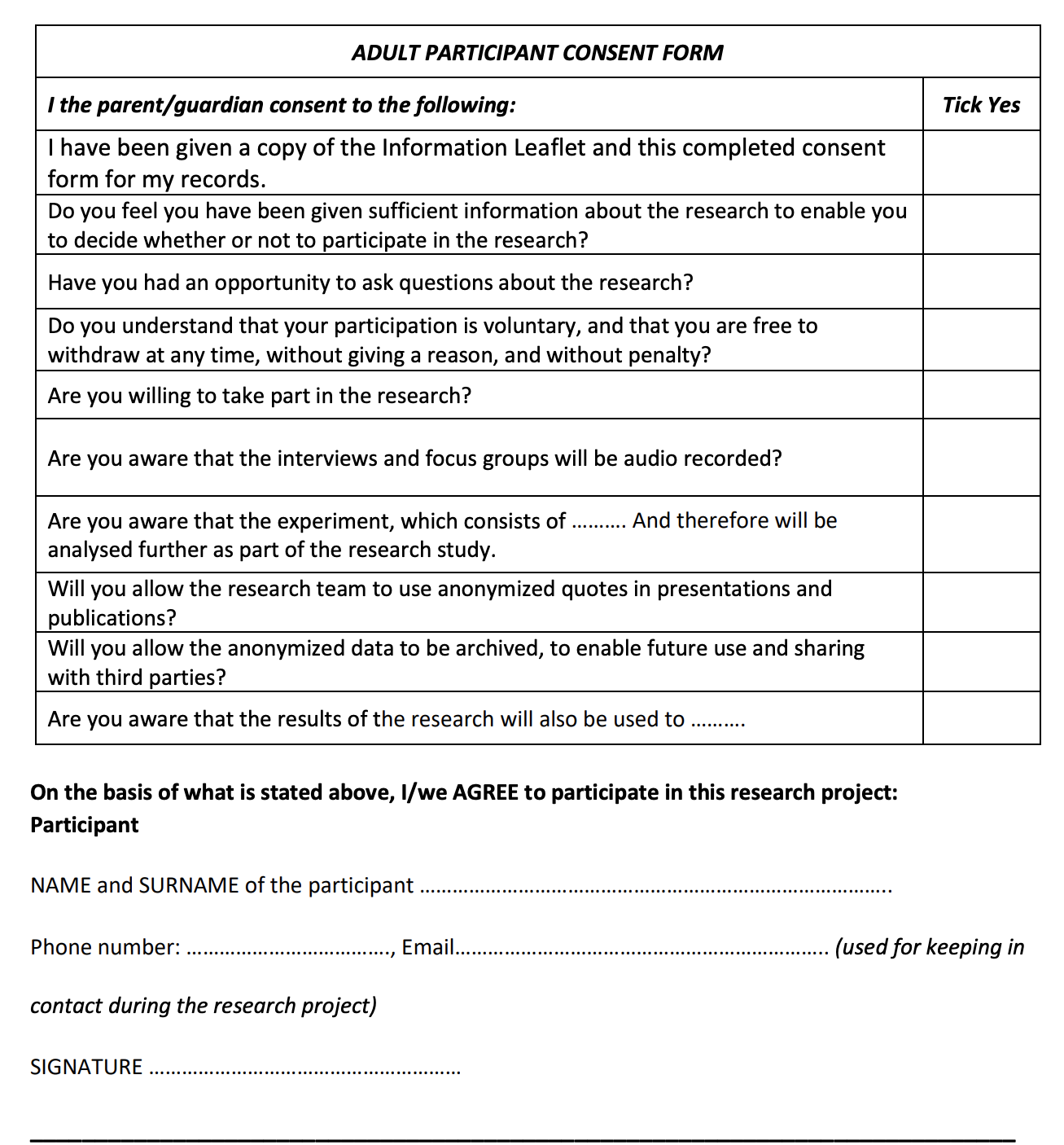


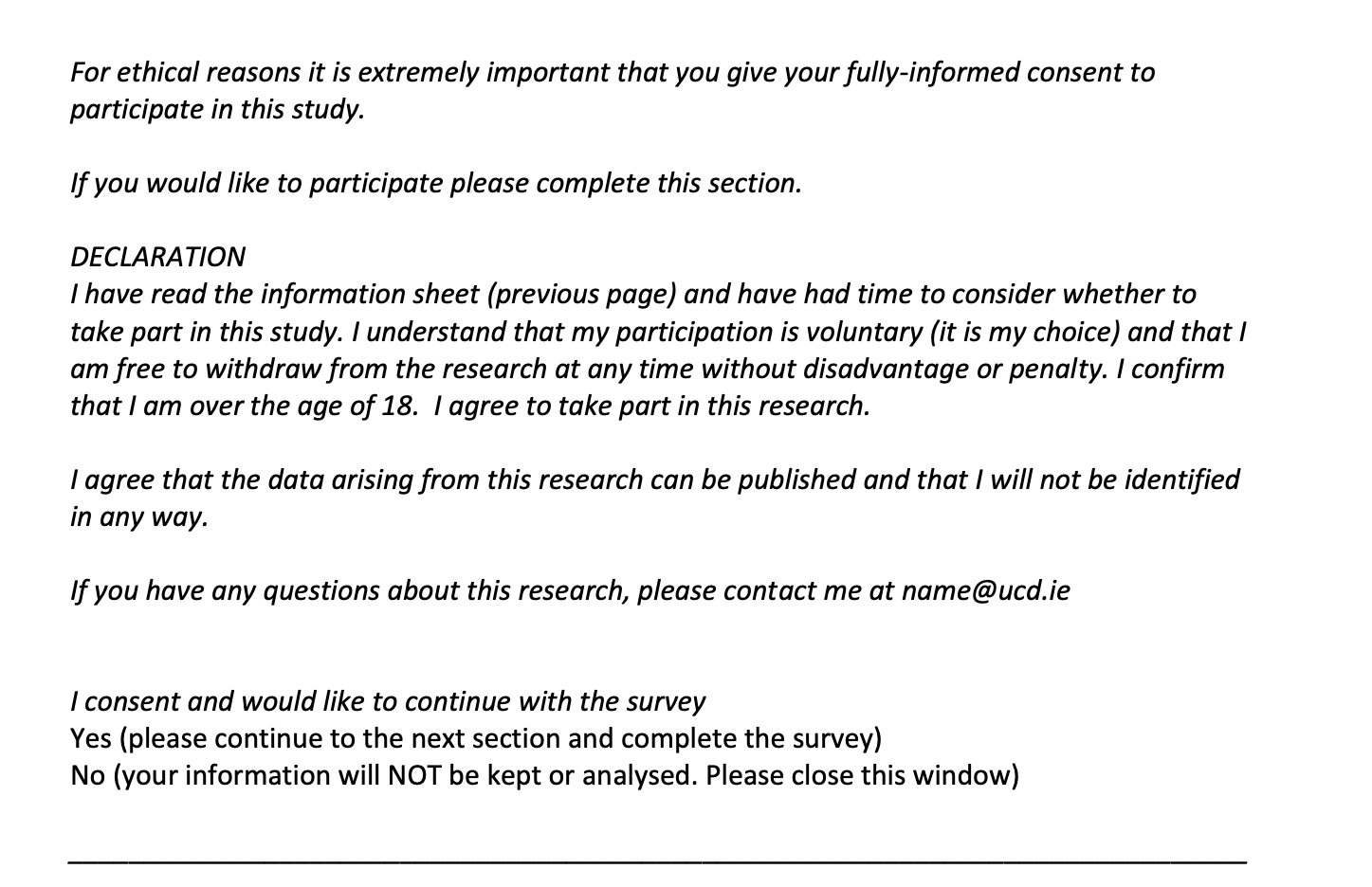


*WEMWBS Scale for Mental Well-being*


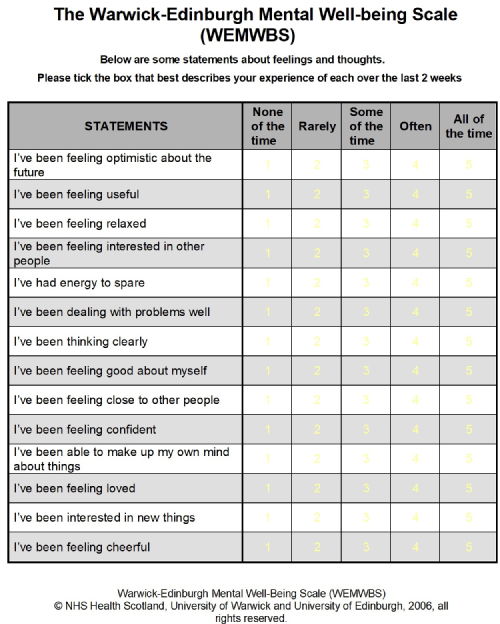


*William’s scale for measuring online and offline social capital.*


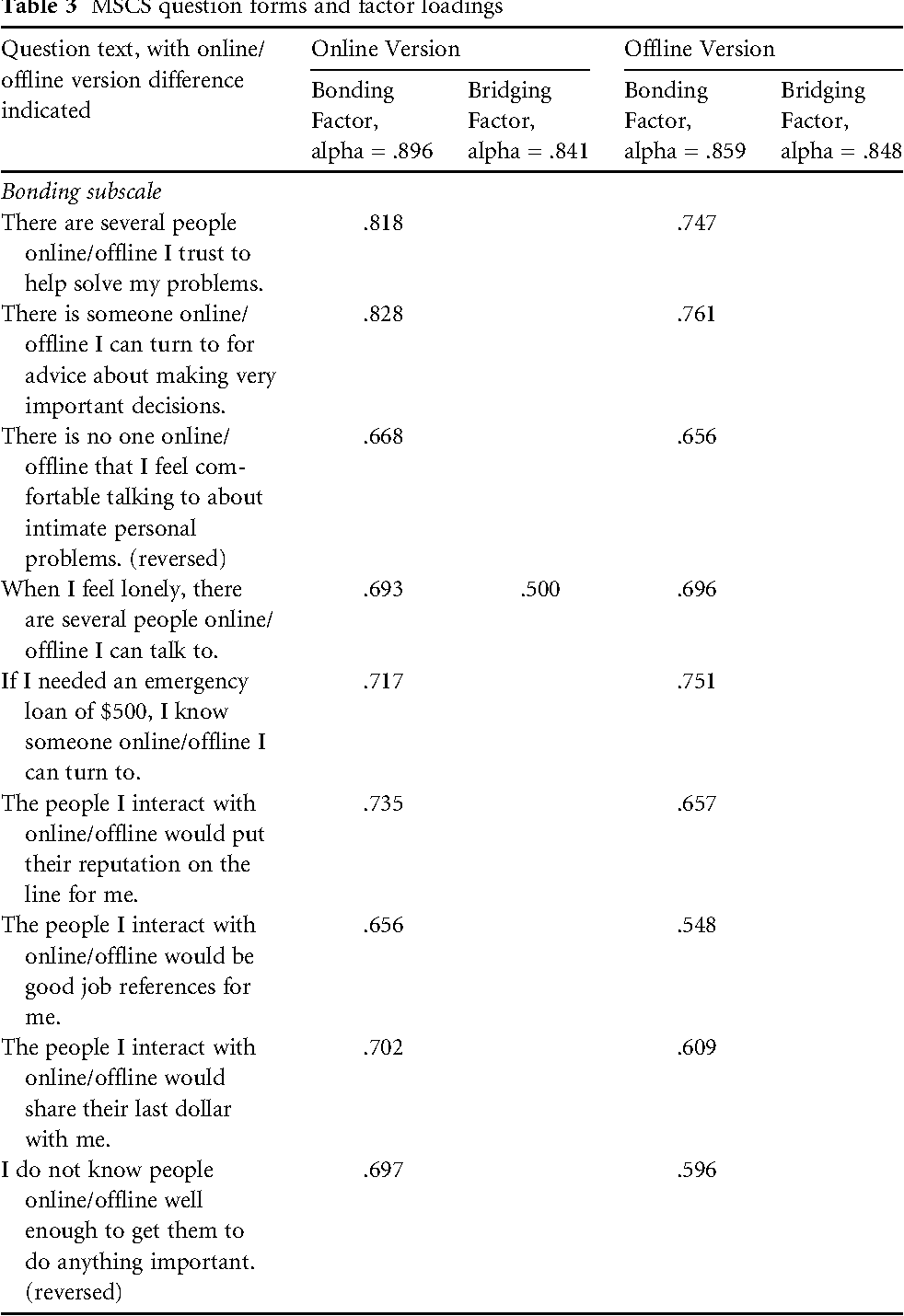

Supplement: Supplementary file 1 [file Table1.docx]
